# Clinician documentation of social circumstances of older and younger women receiving chemotherapy for early breast cancer

**DOI:** 10.1093/oncolo/oyaf357

**Published:** 2025-10-22

**Authors:** Suniti Mohan, Allison M Deal, Wenqing Zhu, Alexis C Wardell, Allison Ross, Kirsten A Nyrop, Hyman B Muss

**Affiliations:** Campbell University School of Osteopathic Medicine, Buies Creek, NC 27506, United States; Lineberger Comprehensive Cancer Center, University of North Carolina at Chapel Hill, NC 27599, United States; Gillings School of Global Public Health, University of North Carolina at Chapel Hill, NC 27599, United States; Lineberger Comprehensive Cancer Center, University of North Carolina at Chapel Hill, NC 27599, United States; Lineberger Comprehensive Cancer Center, University of North Carolina at Chapel Hill, NC 27599, United States; Lineberger Comprehensive Cancer Center, University of North Carolina at Chapel Hill, NC 27599, United States; Division of Oncology, School of Medicine, University of North Carolina at Chapel Hill, NC 27599, United States; Lineberger Comprehensive Cancer Center, University of North Carolina at Chapel Hill, NC 27599, United States; Division of Oncology, School of Medicine, University of North Carolina at Chapel Hill, NC 27599, United States

**Keywords:** social support, breast cancer, clinician notes

## Abstract

**Background:**

The Institute of Medicine (IOM) recommends that essential aspects of a patient’s sociodemographic, psychological, and behavioral characteristics be documented in the electronic health record (EMR).

**Patients and Methods:**

For this study of women receiving chemotherapy for early breast cancer (Stages I-III), EMR clinician notes were queried with regard to documentation of the patient’s current (1) living situation, (2) caregiver responsibilities, and (3) accompaniment during chemotherapy. Descriptive statistics for patient sociodemographic and tumor characteristics, and clinician-reported social circumstances were reported for older and younger patients and compared between two age groups using Fisher’s exact tests for categorical variables and *t*-tests for continuous variables.

**Results:**

The sample includes 104 women aged 65 or older (range 65-83; 17% Black) and 250 under age 65 (range 23-64; 22% Black). Mean number of comorbidities was 3.7 (range 0-8) among older patients and 1.7 (range 0-9) among younger patients (*P* < .0001). There were no significant inter-group differences in breast cancer stage or phenotype. Clinician notes affirmatively documented that the patient was living with someone (70% older/85% younger; *P* = .002), the patient had caregiver responsibilities (12% older/44% younger; *P* < .0001) and was accompanied by someone during chemotherapy (79% older/89% younger; *P* = .02).

**Conclusion:**

Clinician notes pertaining to younger patients as compared to older patients more often provided affirmative and specific documentation that the patient was living with someone, a caregiver for someone, and accompanied by someone during the chemotherapy infusion visit. These three factors are important to document and monitor in patients of all ages as they can impact treatment experience and quality of life during and after chemotherapy.

Implications for Practice Social isolation and low social support are important determinants of health outcomes and mortality, especially in older patients. This study investigated the extent to which clinician notes in the electronic medical record of women with early breast cancer recorded the patient’s marital status and living situation, ongoing caregiver responsibilities, and whether they were accompanied by anyone during a chemotherapy infusion visit. We report that clinician notes pertaining to younger patients as compared to older patients more often provided affirmative and specific documentation that the patient was living with someone, was a caregiver, and was accompanied during the chemotherapy infusion visit.

## Background

Social isolation and low social support have been identified as important determinants of health outcomes and mortality,^1^^,^^2^ especially in older adults.^3^^,^^4^ In a study of adults aged 65 and older, 12-year mortality risk was 23% higher among those having low social support.^5^ And, in a meta-analysis of studies measuring the impact of social relationships on mortality at all ages, there was 50% higher likelihood of survival among adults with strong social relationships as compared to those having weaker levels of support (OR 1.50, 95% CI 1.42-1.59).^6^

Specifically with regard to cancer, in a study of social influences on clinical outcomes in 168 women with ovarian cancer,^7^ women with lower social attachment had a median survival time of 3.35 years as compared to 4.70 years among women with higher social attachment. In a study of quality of life after breast cancer diagnosis,^8^ social isolation (lower scores for social networks and social support) was associated with significantly lower physical, functional, social and emotional well-being and worse lingering breast cancer symptoms. In study of older women with breast cancer, lower social support increased the odds for accelerated declines in social, physical, and cognitive scales measuring quality of life.^9^

In light of growing evidence of the impact of social isolation and social support on health outcomes and mortality, the Institute of Medicine (IOM) recommended that certain sociodemographic, psychological, and behavioral factors be captured in the electronic health record (EMR),^10^ including the individual-level social relationship domain of “social connections and social isolation.” As a measurement tool, the report recommended NHANES III social interaction queries such as marital status/living with someone, interactions with other people (talking or meeting with family, friends, neighbors), attending church or religious services, and belonging to a club or organization (religious group, union, athletic club, school group).

In the current study, we conducted a retrospective review of the electronic medical records (EMR) of women with early breast cancer to extract clinician notes pertaining to the patient’s marital status and living situation, ongoing caregiver responsibilities while they received chemotherapy, and whether they were accompanied by anyone during a chemotherapy infusion visit. All patients in our analysis were offered and received chemotherapy treatment. Our underlying premise is that social support in the home may be a consideration in the clinician’s determination of the patient’s “fitness” for chemotherapy,^11^^,^^12^ and therefore, mentioned in clinician notes. Having versus not having someone to accompany the patient at chemotherapy infusions may also be a consideration in the clinician’s decision to offer chemotherapy and the patient’s decision to accept chemotherapy^12^ and similarly noted in the EMR. And clinician queries about the patient’s caregiver responsibilities during chemotherapy can provide further context for the patient’s living circumstances that may impact treatment experience and quality of life.

## Methods

### Patient sample

Patients in this study were enrolled in one of three studies that investigated a home-based walking intervention during chemotherapy for Stages I-III breast cancer—women aged 21 to 64 years (NCT02167932), aged 65 or older (NCT02328313), and aged 21 or older (NCT03761706). All patients received chemotherapy at the North Carolina Cancer Hospital in Chapel Hill and UNC Rex Healthcare in Raleigh, North Carolina, and all study participants were asked to engage in moderate self-directed walking throughout their chemotherapy treatment. The studies were approved by the University of North Carolina Institutional Review Board and the UNC Lineberger Comprehensive Cancer Center Protocol Review Committee. The enrollment period was from 2014 to 2022. All participants provided written informed consent.

### Measures

#### Electronic medical record

Medical charts of study participants were retrospectively reviewed for medical oncology notes from MDs, Nurse Practitioners (NPs), Physician Associates (PAs), oncology clinic nurses, and infusion nurses throughout the duration of chemotherapy. The entire provider note was read, although the primary focus was social history notes with regard to the patient’s *living situation* at breast cancer diagnosis—living alone (yes/no) and the person with whom the patient was living (spouse/partner, parent/s, adult child/children, young child/children, sibling/s, and other). Second, we searched clinician notes for the patient’s *caregiver status* at diagnosis—was the patient a caregiver for someone (yes/no) and for whom (spouse/partner, parent/s, adult child/children, young child/children, sibling/s, and other). The search was not limited to the specific wording of “caregiver” but instead all notes were read for evidence of family members or other significant persons who accompanied the patient during a clinic or infusion visit. Third, clinician notes were reviewed for comments regarding whether (yes/no) and by whom the patient was accompanied during a chemotherapy infusion visit.

A “yes” entry was entered into our database when the clinician specifically noted the identity of a person with whom the patient was living, for whom the patient was a caregiver, or who accompanied the patient during an infusion visit. EMR data were also extracted with regard to the patient’s breast cancer stage^13^ and phenotype, surgery prior to chemotherapy, chemotherapy treatment plan, and comorbidities at breast cancer diagnosis.

### Statistical considerations

Descriptive statistics for patient sociodemographic and tumor characteristics, and clinician-reported social circumstances were reported for older and younger patients and compared between the two age groups using Fisher’s exact tests for categorical variables and *t*-tests for continuous variables. *P*-values were two-sided with a significance level of *P* = .05. All analyses were conducted in SAS version 9.4 (SAS Institute, Cary, NC, United States).

## Results

### Study participants

The sample (Table 1) includes 104 women aged 65 or older (mean age of 69, 17% Black or 1% other race), and 250 women in the under age 65 group (mean age 49, 22% Black or 7% other race). Average number of comorbidities was lower in younger (1.7, range 0-9) as compared to older women (3.7, range 0-8; *P* < .0001). There were no significant differences between the age groups with regard to tumor characteristics, type of surgery, or receipt of radiation treatment. With regard to chemotherapy regimens, 26% of older women as compared to 46% of younger women received anthracycline based chemotherapy (*P* = .0003).

**Table 1.** Study participants (*N* = 354).

Variable (EBC)	Age >= 65	Age < 65	*P* value
	*N* = 104	*N* = 250	
Age (mean, SD)	69 (4.3)	49 (9.3)	<.0001
Range	65-83	23-64	
Race			
White	85 (82%)	178 (71%)	.03
Black	18 (17%)	55 (22%)	
Other	1 (1%)	17 (7%)	
Comorbidities – mean (SD)	3.7 (2.2)	1.7 (1.8)	<.0001
Range	0-8	0-9	
**Breast cancer diagnosis and treatment (EBC)**			
Breast cancer stage			
I	38 (37%)	84 (34%)	.78
II	46 (44%)	110 (44%)	
III	20 (19%)	56 (22%)	
Subtype			
HR-/Her2-	33 (32%)	67 (27%)	.51
HR-/Her2+	8 (8%)	27 (11%)	
HR+ /Her2-	41 (40%)	112 (45%)	
HR+ /Her2+	22 (21%)	44 (18%)	
Surgery			
None	1 (1%)	3 (1%)	.09
Lumpectomy	64 (61%)	123 (49%)	
Mastectomy	39 (38%)	124 (50%)	
Radiation			
No	23 (22%)	56 (22%)	1.00
Yes	81 (78%)	194 (78%)	
Chemotherapy regimen			
Anthracycline-based	26 (25%)	116 (46%)	<.0001
Not anthracycline based	76 (73%)	134 (54%)	
No information	2 (2%)	0 (0%)	

### Social circumstances noted by clinicians in the EMR

Findings from a review of clinician notes are summarized in Table 2. Younger as compared to older women were more likely to be noted in EMR notes as living with someone specific (85% younger vs 70% older; *P* = .002). Older women were more likely to live only with spouse partner (78% older vs 37% younger; *P* < .0001).

**Table 2.** Social support history recorded in clinician notes.

Variables	Age >= 65	Age < 65	*P* value
	*N* = 104	*N* = 250	
**Living situation**			
Living with someone	73 (70%)	213 (85%)	.002
Person with whom the patient is living			
Only spouse/partner	57 (78%)	78 (37%)	<.0001
Spouse/partner and young children	0 (0%)	66 (31%)	
Spouse/partners and others	5 (7%)	29 (14%)	
No spouse but with others	11 (15%)	40 (19%)	
**Patient’s caregiver status**			
Patient is caregiver for someone else	12 (12%)	109 (44%)	<.0001
Patient is caregiver for			
Young children	0 (0%)	91 (84%)	<.0001
Others	12 (100%)	18 (16%)	
**During chemotherapy accompaniment**			
During chemotherapy, patient is accompanied	82 (79%)	223 (89%)	.02
Accompanied			
Only spouse/partner	26 (33%)	87 (39%)	.33
Spouse/partner and young children	0 (0%)	5 (2%)	
Spouse/partners and others	23 (29%)	63 (28%)	
No spouse/partner but with others	31 (39%)	67 (30%)	

Younger women were more likely than older women to be a caregiver for someone (44% younger vs 12% older, *P* < .0001), especially for young children (84% younger vs 0% older; *P* < .0001).

Younger patients were more likely than older women to be noted in the EMR as accompanied by a specific person during chemotherapy infusion (89% younger vs 79% older; *P* = .017). Exactly who accompanied the patient did not differ between age groups (*P* = .33). Even when not specifying who accompanied the patient during her infusion visit, there were at time other notes in the EMR such as “she has social support” from sister, close knit family, family nearby, nephew, church, or friends, or “she prefers to come alone due to other family demands during weekdays.”

## Discussion

This study aimed to investigate whether and how oncology clinicians noted essential social history factors of older as compared to younger women with early breast cancer who were scheduled for chemotherapy. We found significantly more instances of specific notations about the social circumstances of younger patients as compared to older patients—noted as “yes” she was living with specific person/persons, “yes” she was a caregiver for specific person/persons, and “yes” she was accompanied by specific person/persons during chemotherapy infusion.

Our overall conclusion is that EMR documentation of the social circumstances of women receiving chemotherapy for early breast cancer is generally good and informative, but there is also room for improvement. For example, a specific notation in the EMR that a patient is “living alone” is as important as an affirmative notation of the person/persons with whom the patient is living, because it provides insights as to the potential availability of someone on hand to help the patient with treatment side effects or getting to clinical appointments. The small percent of older patients with positive affirmation that she is the caregiver for someone (11%) may be an issue of interpretation for both patients and clinicians, especially within a dyad of elderly persons where they organically care for each other but do not self-identify as a caregiver.^14^ The 21% of older patients where no one is specifically mentioned as accompanying them during a chemotherapy visit should be concerning, as it may indicate low social support (beyond “living with someone”) when the patient has difficulties tolerating her chemotherapy treatment.

A limitation of our study is that the absence of specific information in the EMR regarding individuals with whom the patient is sharing her home or for whom she is a caregiver or who accompanied her to the infusion visit does not necessarily mean that the patient lives alone, is not a caregiver or was unaccompanied. It only means that this important information regarding a patent’s social history was not proactively noted by clinicians so that it can be monitored during the course of her treatment and thereafter. This is a limitation of retrospective chart reviews, which also includes using data (clinician notes) that were not written for research purposes. A second limitation is that this is a single-institution study and not generalizable to other settings or the larger population of women with breast cancer in the United States or elsewhere. A further limitation is that our notes review may have missed other essential personnel such as social workers. And we acknowledge that our chosen focus on patients receiving chemotherapy precluded a comparison with patients who did not receive chemotherapy whose health outcomes may be similarly impacted by social isolation and low social support.

In our analysis, we grouped “spouse” with “partner.” The literature is already well developed regarding marital status and breast cancer outcomes.^15^^,^^16^ A recent meta-analysis concluded that unmarried women with breast cancer had 22% worse cancer-specific mortality and 20% worse overall survival for reasons that included 28% greater risk for later stage diagnosis.^17^ Marriage appears protective for married women as compared to divorced, single or widowed patients,^18^ although this can vary by race and tumor type.^19^ To the extent the patient is willing to share her living circumstances, it is particularly informative for the EMR to note if she is single, widowed, or divorced as well as whether she is living alone.^20^^,^^21^

At all ages, caregiver responsibilities can be a burden.^22^ NCI has estimated that over 30% of women with breast cancer under age 54 have children under age 18.^23^ It is important for clinicians to stay abreast of the ability of these younger patients to juggle their breast cancer treatment and management of treatment side effects with parenting responsibilities and other concerns.^24–26^ At the other end of the age spectrum, women have on average 5.9 years higher life expectancy as compared to men (year 2021), with age gaps varying by race and ethnicity^27^ and social determinants of health.^28^ Patient-centered queries about marital status and “living alone” should also include questions about the health of the spouse/partner and living arrangement, for insights as to whether the woman with cancer (1) is living with someone who can care for her or (2) has extensive caregiver responsibilities for others that may impact her treatment experience and recovery.

Social support for the patient and its negative corollary social isolation need to be of concern to the treatment team as they consider treatment options and likely outcomes. While some patients decline to discuss or do not volunteer their social circumstances, the social isolation, and loneliness epidemic is of growing concern in the clinic setting.^29^ The epidemic is at all ages^30^ but especially so among the elderly.^31^ It is too much to ask oncology clinicians to delve into these larger issues affecting health and wellness, but doctors, physician associated, nurse practitioners and other personnel treating adults with cancer need to feel comfortable asking patients basic questions about their social circumstances so that they may identify and monitor individual patients with deficits in social support in a timely manner. The EMR should have reminders for clinicians to revisit social history queries at every visit throughout chemotherapy as circumstances may change during the months of treatment and impact quality of life in survivorship. Follow-up visits are also an opportunity to update social histories using a template that reminds clinicians who is considered a major caregiver and suggests review of social work consults if done. It is an opportunity for further research to investigate with clinicians and EMR programmers the most helpful format for social support and social isolation documentation and periodic reviews to ensure this information is up to date and acted on when appropriate.

## Data Availability

There is no database to share. Data was collected through a review of electronic medical records and recorded in author notes.
